# Predictors of maternal near miss among women admitted in Gurage zone hospitals, South Ethiopia, 2017: a case control study

**DOI:** 10.1186/s12884-018-1903-1

**Published:** 2018-06-26

**Authors:** Abebaw Wasie Kasahun, Wako Golicha Wako

**Affiliations:** Department of Public Health, College of Health Sciences and Medicine, Wolkite University, Wolkite, Ethiopia

**Keywords:** Maternal near miss, Healthcare delay, Severe maternal morbidity, South Ethiopia

## Abstract

**Background:**

Maternal mortality and morbidity remain unacceptably high in developing countries. Behind every maternal death, many other women suffered from acute and chronic obstetric complications. Women who survive severe acute maternal morbidities/near miss have many characteristics in common with maternal death events particularly on risk factors. Ethiopia is among countries with high maternal mortality and morbidities in sub-Saharan Africa. However there is scarce evidence on risk factors of severe acute maternal morbidities in Ethiopia. Therefore this study aimed to identify predictors of maternal near miss among women admitted in Gurage zone hospitals, south Ethiopia, 2017.

**Methods:**

Hospital based case control study was conducted to assess predictors of maternal near miss among women admitted in five hospitals of Gurage zone, South Ethiopia. Data of 229 (77 cases and 152 controls) women were included in the analysis. Cases were women admitted due to severe acute maternal morbidity while controls were women admitted for normal labor or women admitted due to mild to moderate obstetric complications. Cases were identified by validated-disease specific criteria. Then, two controls were selected for each verified case using lottery method among eligible women.

Data were collected using interviewer administered questionnaire and reviewing patients’ records. Data were entered using Epi Info 7 and analyzed by SPSS 21. Multivariable logistic regression analysis was done to identify independent predictors of maternal near miss.

**Result:**

Majority of cases were admitted due to dystocia (57.1%) and obstetric hemorrhage (26%). The median first delay (delay to seek health care) among cases and controls was six and 4 h respectively. Prior history of cesarean section {AOR 7.68, 95%CI, 3.11–18.96}, first delay {AOR 2.79, 95%CI, 1.42–5.50}, and being referred from other health facilities {AOR 7.47, 95% CI, 2.27–24.51} were independent predictors of maternal near miss.

**Conclusions:**

Prior history of cesarean section, being referred from other health facilities and first delay were factors associated with maternal near miss. Timely health care seeking behavior of women is uncommon in the study area. Therefore primary health care programs need to enhance the existing efforts to improve timely health care seeking behavior of women.

## Background

Maternal mortality and morbidity remain unacceptably high in developing countries, where 99% of maternal death occurs. The risk of maternal death is 1 in 41 and 1 in 3300 live births in developing and developed countries respectively. For every woman who dies 20 more women experience acute and chronic complications [1, 2].

Studying maternal mortality is increasingly difficult due to its rare occurrence. Consequently, conducting population based study to identify risk factors for maternal death requires long time and huge resource [3]. Women who survive severe obstetric complications have many characteristics in common with maternal death events particularly on risk factors. Recognizing this fact, World Health Organization (WHO) called for increased study of maternal near miss [4]. Maternal near miss is also called severe maternal morbidity [5], hence these terms are used interchangeably.

Maternal near miss is a condition in which a woman nearly dies from complications of pregnancy or childbirth within 42 days of termination of the pregnancy regardless of location or duration, but survives either due to the care she receives or due to chance. Maternal near miss is more valuable indicator for analysis of obstetric care than maternal mortality [6]. It has higher incidence rate than maternal mortality. Some studies reported maternal near miss is 15 times more frequent than maternal death [7] and another study in low resource setting showed that maternal near miss occurred 26 times more frequent than maternal death [6]. Thus, maternal near miss study allows rigorous quantitative analysis of factors leading to severe maternal morbidity and maternal mortality. Moreover, it offers a good opportunity for data collection as a woman herself can be a source of information [8].

Prevalence of maternal near miss varies across global regions. It ranges from 0.6 to 14.98% by disease specific criteria and 0.04 to 4.54% by management based criteria. The magnitude of maternal near miss is high among African and Asian middle and low income countries [9]. A systematic review done by WHO also showed that prevalence of severe maternal morbidity (near miss) varies between 0.80–8.23% among studies which used disease specific criteria and 0.01–2.99% among studies that used management based criteria [10]. According to WHO, prevalence of severe maternal outcomes (maternal death and maternal near miss) is expected to be 7.5 per 1000 live births [11].

Previous researchers have shown that prior history of cesarean section, primipara, women who gave birth three and more times, age greater than 35 years, low socio economic status, lack of transport, rural residence, previous history of anemia, hindrance of family members in seeking timely care, chronic medical disorders, delay to reach or access care, delay in receiving care and non-adherence to antenatal care are significant determinants of maternal near miss [4, 5, 12–15].

Reducing maternal mortality is one of the Sustainable Development Goals (SDGs) launched to be achieved by the year 2030. Global countries have pledged to reduce maternal mortality ratio below 70 per 100,000 live births for all countries by the year 2030 [16]. Identifying predictors of maternal near miss would contribute for realization of the global target of maternal mortality reduction in one or another way.

Ethiopia is among countries with the highest maternal mortality rate in the globe [17]. In Ethiopia, 20,000 women die each year during pregnancy, child birth and postpartum period [18]. According to 2016 Ethiopian Demographic and Health Survey (EDHS) report, maternal mortality ratio was 412 per 100,000 live births [19]. Beside this high toll of maternal death, many more women suffered from acute and chronic obstetric complications. In order to curb this high maternal mortality and morbidity, identifying the determinant factors is crucial. However studies on determinants of maternal mortality and maternal near miss are scarce in Ethiopia. Even though there are few studies on maternal mortality and maternal near miss, they solely depended on hospital records which hardly capture complete socioeconomic and other factors responsible for maternal near miss. Therefore this study aimed to investigate determinants of maternal near miss among women admitted in Guragie zone hospitals.

### Operational definition

#### Maternal near misses (severe maternal morbidity)

Women who are admitted with either of the following obstetric diagnosis: severe preeclampsia, eclampsia, severe hemorrhage, dystocia (defined in the current study as uterine rupture, impending uterine rupture like prolonged labor with previous cesarean section, and emergency C/S delivery), severe anemia (hgb < 6 g/dl), sepsis (puerperal sepsis, chorioamniotis and septic abortion).

## Methods

### Study area

Gurage zone is one of the administrative zones in South Ethiopia. It has 13 districts and two town administrations**.** Wolkite town is the capital of Gurage zone. It is found 153 km southwest to Addis Ababa, the capital of Ethiopia. According to the 2007 national household census, Gurage zone has total population of 1,279,646, of which 657,568 are women [20]. There are five hospitals (three public and two non-governmental) serving the total population in the zone. Four of the hospitals in the zone are primary hospitals and the remaining one is general zonal hospital. All hospitals provide comprehensive emergency obstetric care services. Additionally there are 72 health centers which provide basic emergency obstetric care services in Gurage zone. All the five hospitals in Gurage zone were involved in the study.

### Study period

The study was conducted from October 2016 to May 2017 among pregnant and postpartum women admitted in five hospitals of Gurage zone, South Ethiopia.

### Study design and study population

Hospital based case control study was conducted among women admitted in five hospitals of Gurage zone, South Ethiopia.

All women who were admitted during pregnancy, labor, or within the first 42 days of postpartum were the study populations. Cases were women admitted with maternal near miss event, and controls were women admitted for normal vaginal delivery or women with mild to moderate obstetric complications. Mild to moderate obstetric complications includes but not limited to premature rapture of membrane, non-reassuring fetal heart beat, hyperemesis gravidarum, placenta previa and abruption placenta with minimal blood loss, retained placenta and mild pre-eclampsia,

Women who were not permanent residents of Gurage zone (lived less than 6 months in the study area) were excluded.

All women who were admitted with maternal near miss during the study period were consecutively included as cases. For each near miss case, two controls admitted within 24 h of case’s admission were selected. Lottery method was employed to recruit controls among eligible women (i.e. women who did not meet criteria for cases) admitted in the same hospital where cases were identified.

### Sample size determination and sampling procedure

Sample size was determined using Epi Info 7 software StatCalc menu for unmatched case control study. The following assumptions were considered to calculate the sample size: power of 80%, confidence level of 95% and control to case ratio of two (2:1). Proportion of maternal near miss among rural women was 11.2% and the odds of developing maternal near miss among rural women was 2.96 times higher compared to the urban women [21]. Total sample size required was 230 women (77 cases and 153 controls).

The last 6 months’ obstetric case management report of each hospital was used to determine obstetric client/patient flow rate of respective hospitals. Then sample size was proportionally allocated to each hospital in Gurage zone based on expected obstetric client/patient flow rate.

Validated disease-specific approach with five diagnostic criteria [severe hemorrhage, hypertensive disorders (severe pre-eclampsia, eclampsia), dystocia (uterine rupture, impending uterine rupture like prolonged labor with previous C/S, emergency C/S), sepsis and severe anemia] was used to identify maternal near miss cases [4]. Cases were identified using Patient cards, admission log books and operation theatre log books. Identified cases were verified by senior physicians working in the study hospitals. Finally, two controls were selected for each verified case using lottery method among eligible women (i.e. women who did not meet criteria for cases) admitted within 24 h of case’s admission.

### Data collection tool and procedure

Data were collected using close ended WHO standard multi-country survey questionnaire developed for maternal near miss study with minor modification to fit the local context [4, 5, 22]. Organ dysfunction assessment part of the WHO near miss questionnaire was not used in this study. This part of near miss assessment questionnaire requires advanced laboratory investigations which are not routinely available in the study setting. Data collectors were junior public health professionals, clinical nurses and midwife nurses. They were recruited from staff of the study hospitals. Data were collected by interviewing women at admission or some time latter during their stay at hospital depending on the patient/client clinical condition. Surrogates of admitted women were also interviewed when the clinical condition of women under study did not enable them to speak easily. Data which cannot be obtained by interview like diagnosis of obstetric complications, management given and laboratory investigation results were extracted from patient records. Data regarding socio-demographic characteristics of study subjects, information related to delays to seek or receive health care and other background factors were collected by interviewing women or their surrogates. Both Interviewing and data extraction from patient records were done by the same data collectors. Data collection process was supervised by trained general practitioner working for the study hospitals.

The study protocol was approved by institutional ethical review board of Wolkite University. Information regarding the study was orally explained to study participants after they were stabilized from their clinical emergency or obtained the required service. Moreover, one page written summary of study information was given to those women who can read and understand Amharic. Then informed verbal consent was directly obtained from study participants. Consent to participate in the study was requested after a patient is stabilized from state of clinical emergency. All eligible contacted women voluntarily participated in the study. Obtaining verbal consent is the acceptable standard of ethical statement in Wolkite University regarding studies which are not clinical trial or studies which do not require invasive procedures.

Interview was done using structured close ended anonymous questionnaire. Data collected from surrogates were communicated to respective women under study to endorse and/or to add information that surrogates did not know. The participants were informed about their right to totally refuse or not to respond to part of a questionnaire without compromising hospital care.

### Data quality measures

Data collectors and supervisors had given 2 days intensive training on data collection tool including data abstraction template, objective of study, and techniques of data collection. The Principal investigator and supervisors made frequent checks for consistency and completeness of collected data and appropriate corrections were made on the spot.

### Data processing and analysis

Data were entered using Epi Info7 and exported to SPSS version 21 for analysis. Descriptive statistics like frequencies, proportions, median and mean were used to explain important variables in relation to the outcome variable. Chi-square test was used to compare proportion of cases and controls in terms of selected categorical variables. Independent sample t-test was also used to test equality of means for selected continuous variables among cases and controls. First binary logistic regression was done to assess the association of each independent variable with the outcome variable. Then, to control confounding variables, multi -variable logistic regression analysis was done by taking all variables with *p*-value < 0.2 on binary logistic regression into the model at the same time. p- Value of 0.05 or less and adjusted odds ratio (AOR) with 95% confidence interval were used to declare that a variable is independent predictor of the outcome variable. Hosmer-Lemeshow goodness of fit test was conducted to ascertain whether the model was correctly specified or the data conflicted with assumptions made by the model.

## Results

Two hundred thirty women were interviewed and their records were also reviewed. Of the interviewed women, records of 229 (99.5%) women were complete and retained for analysis**.** One respondent’s data were excluded from analysis due to missing of major variables. Therefore the final analysis was based on 229 women’s data (77cases and 152 controls).

### Socio-demographic characteristics of women

The mean age of cases and controls was 28.1 and 27.2 years respectively. However the mean age difference between cases and controls was not statistically significant on independent sample t-test (*p = 0.24*). Other socio-demographic characteristics of women are summarized in Table 1 below**.** There was no significant variation in socio-demographic characteristics of cases and controls (*P*-value > 0.05 for all socio-demographic variables).

**Table 1:** Socio-demographic characteristics of women admitted for obstetric reasons in Gurage zone hospitals, South Ethiopia, 2017 (*N* = 229)

Variable	Category	Cases (*n* = 77)		Controls (*n* = 152)	
		Frequency	Percentage (%)	Frequency	Percentage (%)
Residence	Rural	50	64.9	107	29.6
	Urban	27	35.1	45	70.4
	Total	77	100	152	100
Marital status	Married	76	98.7	151	99.7
	Divorced	1	1.3	1	0.7
	Total	77	100	152	100
Maternal occupation	Housewife	58	75.3	122	81.3
	Merchant	2	2.6	11	7.2
	Employed	7	9.1	7	4.6
	Farmer	8	10.4	7	4.6
	Others	2	2.6	5	3.2
	Total	77	100	152	100
Education status of women	Illiterate	29	37.7	56	36.8
	Read and write only	9	11.7	25	16.4
	Primary school first cycle	10	13.0	18	11.8
	Primary school second cycle	12	15.6	36	23.7
	Secondary school	9	11.7	8	5.3
	Diploma and above	7	9.1	9	5.9
	Total	77	100	152	100

### Reasons of admission of cases and controls

One hundred thirty controls (85.5%) were admitted for normal labor and the remaining 22 (14.5%) controls were admitted due to obstetric complications not progressed to maternal near miss. Dystocia was the most common reason for admission of cases. It accounted for 57.1% of all maternal near misses. Other severe maternal morbidity distributions among women admitted in Gurage zone hospitals are summarized in Table 2**.**

**Table 2:** Severe maternal morbidity distributions among women admitted in Gurage zone hospitals, 2017

Cause of near miss	Frequency	Percent
Dystocia	44	57.1%
Hemorrhage	20	26.0%
Hypertensive disorders	13	16.9%
Severe Anemia	7	9.1%
Maternal infections	3	3.9%

NB. One admitted case may have more than one cause of near miss so that the sum of percentages of near miss causes could exceed 100% or the sum of frequency of cases could exceed 77

### Reproductive history of women

Ninety eight percent of controls were pregnant at the time of admission. Of these 94% were at term, 3.3, 0.7% were preterm and post term respectively. Similarly 97.3% of cases were pregnant at the time of admission. Of these, 70.1% were at term, 19.5, 3.9% were preterm and post term respectively. On the other hand, four controls and five cases were admitted during postpartum.

Nearly all controls (98%) gave birth at hospital and the remaining 2% of controls gave birth at home. On the other hand 87% of cases gave birth at hospital, 5.2% at health center and, 2.6% at home. Majority of controls (94.7%) gave birth by spontaneous vaginal delivery, followed by cesarean section (2.6%) and instrumental delivery (2.6%), whereas majority of cases (58.4%) gave birth by emergency cesarean section. Other reproductive histories of cases and controls are summarized in Table 3 below.

**Table 3:** Reproductive history of women admitted in Gurage zone hospitals, 2017, (*N* = 229)

Variable	Category	Cases (*n* = 77)		Controls (*n* = 152)		X^2^-Test
		Frequency	Percentage (%)	Frequency	Percentage (%)	
History of ANC visit	Yes	62	79.2	129	84.9	0.24
	No	15	20.8	23	15.1	
	Total	77	100	152	100	
Prior history of C/S	Yes	23	29.9	11	7.2	< 0.001
	No	54	70.1	141	92.8	
	Total	77	100	152	100	
History of female genital cutting	Yes	63	81	124	81.6	0.48
	No	14	19	28	18.4	
	Total	77	100	152	100	
Maternity status at admission	Pregnant	72	97.3	148	98	
	Postpartum	5	2.7	4	2	
	Total	77	100	152	100	
Gravidity	Primigravida	21	27.3	43	30.9	0.21
	Multigravida	56	72.7	109	69.1	
	Total	77	100	152	100	

*ANC* Antenatal care, *C/S* Cesarean section

### First delay (delay before seeking health care)

One hundred twenty eight (84.2%) controls were self-referred while the remaining 24 (15.6%) controls were referred from other health facilities for further diagnosis and management. Nearly half of cases, 37 (48.1%) had referred from other health facilities and the remaining 40 (51.9%) cases were self-referred.

The median time of delay to seek health care among cases and controls was 6 and 4 h respectively. Shortage of money and not considering symptoms as severe were the dominant reasons for first delay among controls while lack of transportation and not considering the symptoms as severe were prominent reasons for first delay among cases.

### Second delay (delay in reaching to health facility)

The median time taken to reach to the nearest health facility on foot among cases and controls was reportedly 4 and 3 h respectively. Majority of controls (69.7%) used public transport to reach health facilities. About one quarter of controls (26.3%) used ambulance while the remaining controls reach to health facility carried by men or on walk. Public transport is used by 44 (57.1%) cases while, 31 (40.3%) cases used ambulance and the rest cases reach health facility carried by men. The median time taken to get referral for indicated woman was 1 and 2 h among controls and cases respectively.

### Third delay (delay in receiving care)

The median time taken to get clinical interventions among controls was 4.6 h after admission and the median time taken to get clinical interventions among cases was 50 min after admission. The three delays in maternity health care are summarized in Table 4 below.

**Table 4:** Patterns of the three delays for maternal health care among women admitted in Gurage zone hospitals, South Ethiopia, 2017

Variable	Category	Case	Controls	X^2^- test
First Delay	Delayed ≤4 h at home	34	103	0.001
	Delayed > 4 h at home	43	49	
Second delay	take ≤4 h to reach health facility on foot	42	104	0.028
	take > 4 h to reach health facility on foot	35	48	
Third Delay	Treatment delayed ≤2 h after admission	45	2	0.001
	Treatment delayed> 2 h after admission	32	150	

### Determinants of maternal near miss

Multivariable logistic regression revealed that prior history of cesarean section {AOR7.68, 95%CI, 3.11–18.96}, delaying more than 4 h to seek health care {AOR2.79, 95%CI, 1.42–5.50}, and being referred from other health facilities {AOR7.47, 95%CI, 2.27–24.51} were factors significantly associated with maternal near misses.

Women with prior history of cesarean section had 7 times higher odds of developing maternal near miss compared to women with no prior history of cesarean section. Similarly women who delayed more than 4 h to seek heath care had nearly 2.8 times higher odds of developing maternal near miss compared to women who sought health care earlier. Mode of admission was also a significant predictor of maternal near miss. Referred women had 7 times higher odds of developing maternal near miss compared to self-referred women. The overall model fitness (Hosmer-Lemeshow) test showed that the model fitted the data well with *p*-value of 0.12. Predictors of maternal near misses are summarized in Table 5.

**Table 5:** Multivariable logistic regression model for predictors of maternal near misses among women admitted in Gurage zone hospitals, South Ethiopia, 2017 (*N* = 229)

Variable	Category	Cases (*n* = 77)	Controls (*n* = 152)	COR, 95% CI	AOR, 95%CI	*P*-value
Residence	Rural	50	107	1.23 (0.68–2.22)	0.60 (0.33–1.74)	0.14
	Urban	27	45	1		
History of C/S	Yes	23	11	5.22 (2.37–11.49)	7.68 (3.11–18.96)*	< 0.001
	No	54	141	1	1	
Mode of admission	Referred by facility	37	23	4.8 (2.56–8.98)	7.47 (2.27–24.51)*	0.001
	Self-referred	40	128	1	1	
History of ANC	Booked	61	129	1	1	
	Not booked	16	23	1.47 (0.78–1.51)	0.76 (0.33–1.75)	0.52
First delay	Delayed> 4 h	43	49	2.65 (1.51–4.67)	2.79 (1.42–5.50)*	0.003
	Delayed≤4 h	34	103	1	1	
Delay to refer	Delayed > 4 h	24	19	3.17 (1.60–6.26)	1.51 (0.42–5.49)	0.51
	Delayed≤4 h	53	133		1	
Second Delay	Travel > 4 h on foot	35	48	1.80 (1.02–3.17)	0.8 (0.39–1.63)	0.54
	Travel≤4 h on foot	42	104	1	1	

*Statistically significant variables at p-value of < 0.05, *COR* Crude odd ratio*, AOR* Adjusted odd ratio

## Discussion

Prior history of cesarean section, referral from other health facilities and first delay (delay to seek health care) were significantly associated with maternal near misses.

Women who stayed at home for more than 4 h were at higher risk of developing maternal near miss than women who sought health care earlier. This finding is consistent with studies in Nigeria and Morocco [23, 24]. Median time taken to seek health care among cases and controls was six and 4 h respectively. It is noticeable that if minor obstetric complications are not managed in a timely fashion it would progress to severe form of complications. The acute nature of obstetric complication fuels the adverse impacts of delay to seek, reach or receive appropriate health care. Hence, enhancing timely health seeking behavior is imperative to reduce severe obstetric outcomes.

Women with prior history of cesarean section were at higher risk to develop maternal near misses compared to their counterpart. In line with this finding, global survey on maternal near miss reported that prior history of cesarean section was a risk factor for maternal near misses [5]. Studies in Brazil and South Africa also illustrated that prior history of cesarean section is a risk factor for maternal near miss [23, 25–27]. Similarly, a study elsewhere in Ethiopia showed prior history of cesarean section increased odds of maternal near miss in subsequent pregnancies [12]. However a study in Tanzania, indicated that previous Cesarean section is not a risk factor for severe maternal outcomes [28]. Cesarean section carries many risks to women including uterine scar and increased risk of uterine rupture in giving vaginal delivery for subsequent pregnancies. This finding suggests that health care providers should take into account the potential risk of cesarean section while assessing clinical indications for cesarean section. Cesarean section should be done only when there is convincing clinical indications, in other words cesarean section mode of delivery should not be considered for non-medical reasons. Despite associated risks, rate of cesarean section is increasing above WHO recommended level (5–15%). In Addis Ababa, 21.4% of births were given by cesarean section [29]. Hence, non-medical indications of cesarean section should be deterred to reduce the rate to the acceptable level and to reduce health risks associated with cesarean section.

Women who were referred from other health facilities were at higher risk of maternal near misses compared to self-referred women. This finding is in line with a study conducted in Nigeria [30]. This can be due to the fact that more severe obstetric complications are more likely to be referred and lack of transport or too long distance to reach referral facilities would contribute for maternal near miss event. Moreover, delayed referral would contribute to maternal near miss. Improving access to road and other transport facilities, improving referral system and further decentralizing maternity care could reduce maternal near miss events.

Majority of cases were admitted due to dystocia. It represented 57.1% of maternal near misses. Its occurrence is higher compared to findings in Pakistan and Nigeria, where it accounted 14.8 and 23% of maternal near misses respectively [13, 31]. The difference could be due to high prevalence of female genital mutilation in this study area. About 81.7% of the study participants had history of female genital mutilation. Ethiopia has high prevalence of female genital mutilation compared to the aforementioned countries [32, 33]. It is also reported that female genital mutilation is associated with obstetric complications including dystocia, perinatal tear and hemorrhage [34, 35]. The observed difference in occurrence of dystocia might partly be attributable to high prevalence of female genital mutilation in Ethiopia. Furthermore it could be due to delay of life saving interventions which may facilitate development of maternal near miss. The median time elapsed to seek health care among cases and controls were six and 4 h respectively. Women were also expected to walk an average of 3 h to reach to the nearest health facility. The aforementioned scenarios may preclude possibility of managing dystocia before it end up in maternal near miss. Another study in Ethiopia showed that dystocia causes 45% of maternal near miss events [36].

Obstetric hemorrhage was the second most common severe maternal morbidity in the study area. Twenty six percent of cases were admitted due to severe obstetric hemorrhage. There are comparable incidences of hemorrhage associated near misses in Brazil and Turkey [37–39]. Another study in Ethiopia revealed that obstetric hemorrhage and hypertensive disorders of pregnancy (pre- eclmpsia and eclampsia) are the leading causes of maternal near misses [40]. Obstetric hemorrhage is also the second most common cause of maternal mortality in Ethiopia next to obstructed labor [41]. There was also another study which put obstetric hemorrhage as the third most common cause of maternal mortality in Ethiopia [42]. Based on these researches, it can be speculated that maternal near miss is a good proxy variable to study maternal mortality.

Hypertensive disorders of pregnancy caused significant proportion of maternal near misses in this study. About 16.9% of maternal near misses were due to severe pre-eclampsia and eclampsia. Studies conducted in Nigeria, Uganda and Rwanda revealed that hypertensive disorders of pregnancy were responsible for 37.3, 7 and 28.6% of maternal near misses respectively [23, 43, 44]. These variations could be due to the difference in clinical definition for severe pre-eclampsia. Studies which used the most stringent case definition of hypertensive disorders of pregnancy as a cause of maternal near miss may report low incidence and vice versa. Moreover study settings may affect the occurrence of hypertensive disorders of pregnancy. A study in Ghana revealed that urban women have higher incidence of pregnancy induced hypertension compared to rural women [45].

In this study, 9.1% of maternal near misses were due to severe anemia (hgb < 6 g/dl). This finding is lower than incidence of maternal near miss due to severe anemia reported in other studies. In Pakistan, anemia causes 21.2% of maternal near misses; and studies from Nigeria indicate that severe anemia causes 14.6 and 10.7% of maternal near misses [13, 23, 31]. The difference in staple food might contribute for the observed variation in incidence of anemia. In Ethiopia the staple food is iron rich crop called “*Teff*” (*Eragrostis abyssinica*) which is prepared in different edible forms most commonly “*Injera*” (pancake style bread made from “*Teff*”) [46].

### Study limitations

Disease-specific criteria were used to identify maternal near miss events which are less stringent than other clinical criteria; as a result probability of over reporting cases could happen. Verification of cases was done by different physicians so that subjective misclassification of cases and controls cannot be completely ruled out. Information is elicited retrospectively; hence recall bias could have an effect on quality of data. Unmatched case control study design was applied so that cases and controls are not matched with relevant variables to control confounding. It is also difficult to infer the findings of the study to the general population since the study population may not be representatives of the general population.

## Conclusions

Timely health care seeking behavior of women is uncommon in the study area. Consequently, considerable number of women are developing severe acute maternal morbidities that can be easily addressed had it been managed timely. The effect of the aforementioned behavioral problem is fueled by long distance travel to reach health facility, lack of transport and delayed referral to most appropriate health facility. Moreover, women with prior history of cesarean section have shown disproportionate vulnerability to develop maternal near miss in subsequent pregnancies.

Improving timely health care seeking behavior of women is imperative. In current Ethiopian context, Health extension workers are providing health information and other preventive health services to household members by making house to house visit. These efforts need to be enhanced to raise health consciousness of community members. Moreover women who had prior history of cesarean section need special attention from their families and health care providers to proactively mitigate the occurrence of severe obstetric complications.

## Abbreviations

ANC: Antenatal care
AOR: Adjusted odds ratio
C/S: Cesarean section
SPSS: Statistical package for social science
WHO: World Health Organization
